# Supplementary material for: Anthropogenic aerosol and cryosphere changes drive Earth’s strong but transient clear-sky hemispheric albedo asymmetry
Source: Commun Earth Environ. 2022 Sep 12;3(1):206. doi: 10.1038/s43247-022-00546-y (PMC9466336; doi:10.1038/s43247-022-00546-y)
Supplement: Supplementary file 1 — Supplementary Information [file 43247_2022_546_MOESM1_ESM.pdf]

Supplementary Information for "Anthropogenic aerosol and cryosphere changes drive Earth's strong but transient clear-sky hemispheric albedo asymmetry"

Michael S. Diamond[1,2,*], Jake J. Gristey[1,2,3], Jennifer E. Kay[1], & Graham Feingold[2]

1. Cooperative Institute for Research in Environmental Sciences, University of Colorado, Boulder, CO 80309, USA
2. NOAA Chemical Sciences Laboratory, Boulder, CO 80305, USA
3. Laboratory for Atmospheric and Space Physics, University of Colorado, Boulder, CO 80303, USA

*Email: michael.diamond@noaa.gov

Contents

- Supplementary Figure 1: Maps of clear-sky reflection.
- Supplementary Figure 2: Maps of the atmospheric contribution to clear-sky reflection for CERES and the CMIP6 models.
- Supplementary Figure 3: Maps of the surface contribution to clear-sky reflection for CERES and the CMIP6 models.
- Supplementary Figure 4: Zonal differences between CERES and the CMIP6 observations.
- Supplementary Figure 5: Change in hemispheric aerosol asymmetry due to historical emissions of aerosols and their precursors.
- Supplementary Figure 6: Modern and full historical regression slopes for each CMIP6 model.
- Supplementary Figure 7: Emergent constraint for the change in hemispheric aerosol contrast from present-day to pre-industrial based on present-day global mean aerosol optical depth.
- Supplementary Figure 8: Change in surface reflection over the poles in the SSP3-7.0 high-emissions scenario.
- Supplementary Figure 9: Radiative transfer calculations of the clear-sky albedo for a hypothetical "cleaner" Northern Hemisphere.

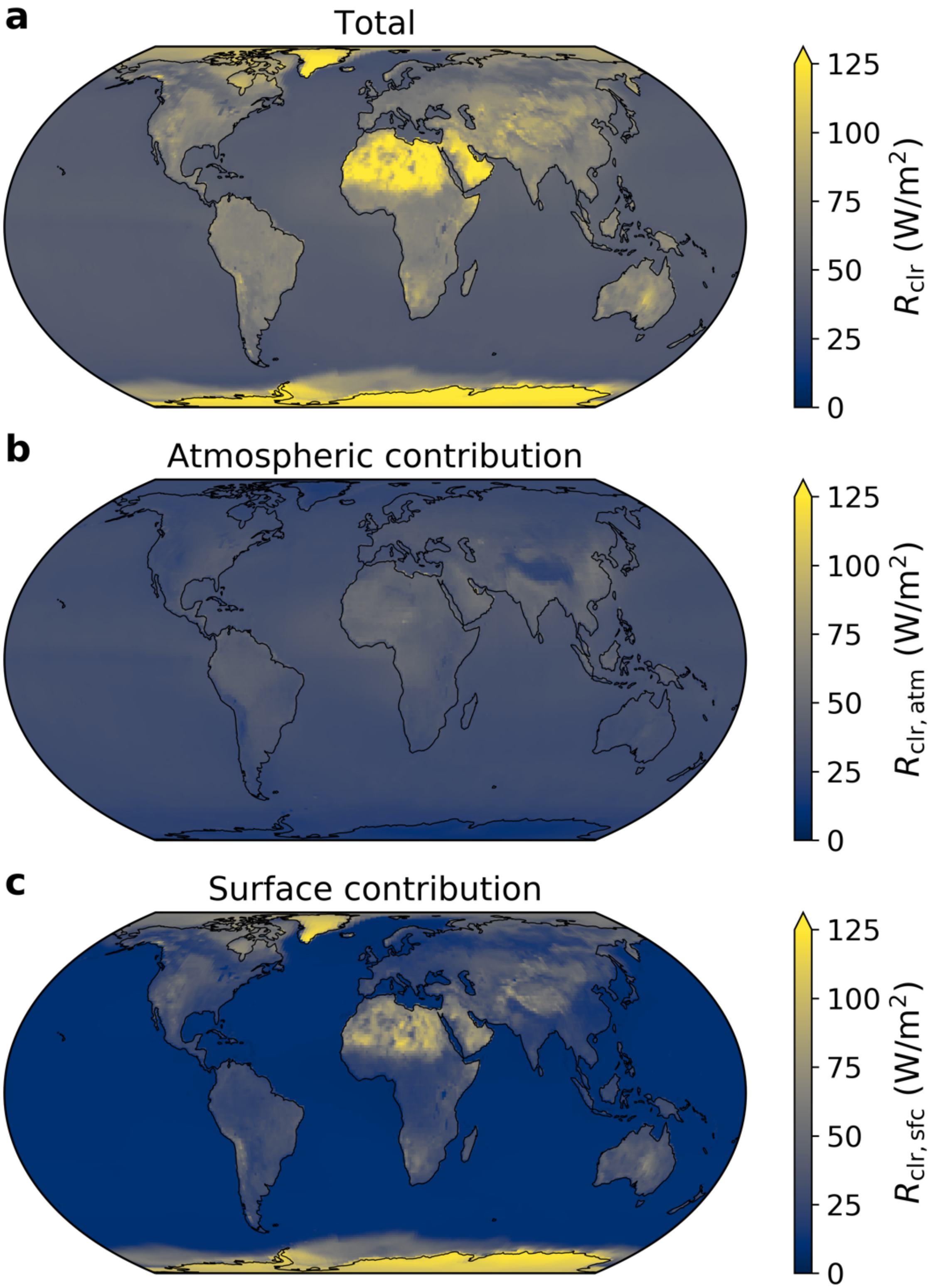

Supplementary Figure 1 | Maps of clear-sky reflection. Total R_{clr} (**a**) and its atmospheric (**b**) and surface (**c**) components are shown globally on an equal-area projection[88,93].

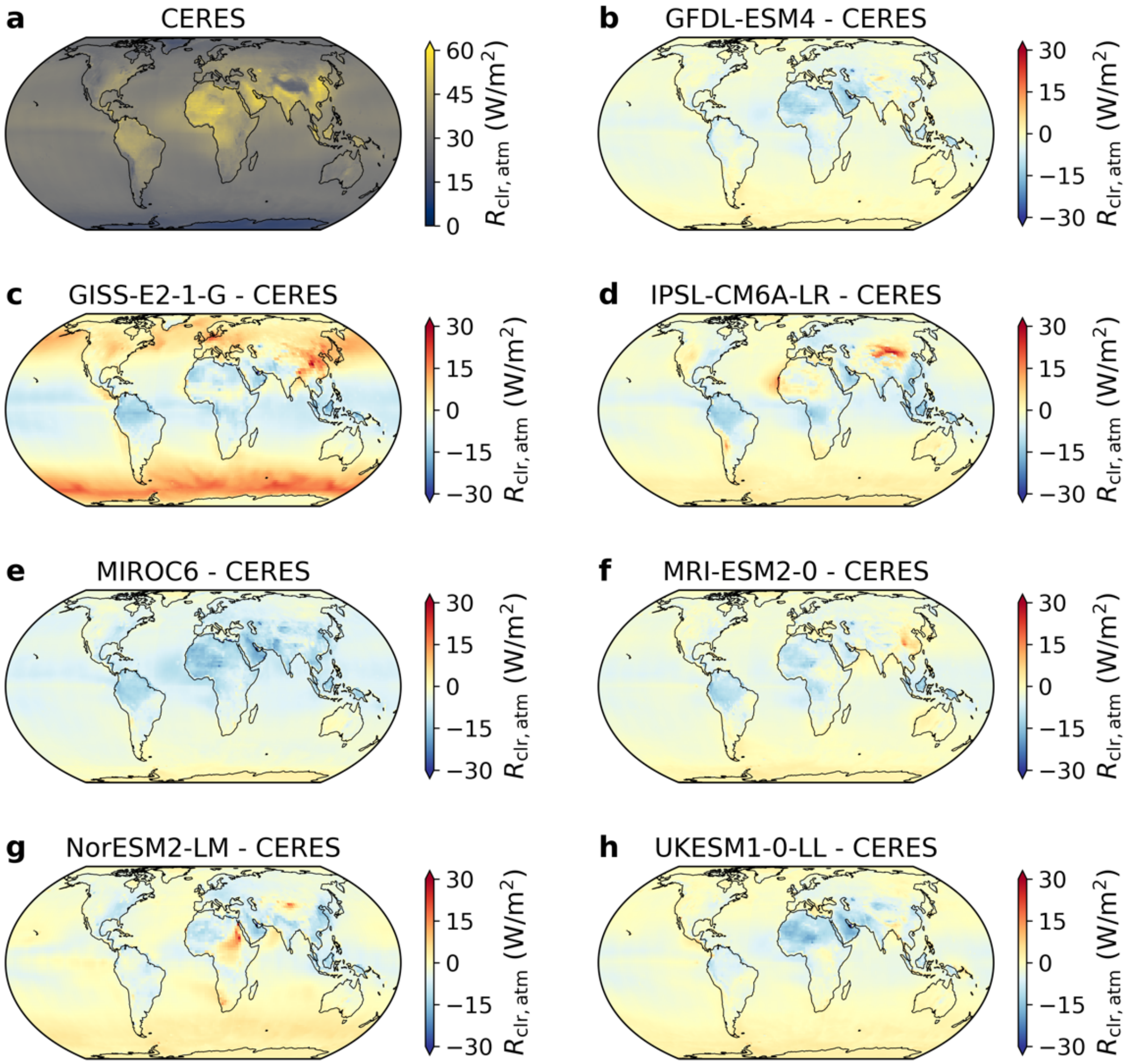

Supplementary Figure 2 | Maps of the atmospheric contribution to clear-sky reflection for CERES and the CMIP6 models. Observed $R_{clr,atm}$ from CERES (**a**) and the difference between the observed value and each of the CMIP6 models analyzed (**b-h**) are shown globally on an equal-area projection[88,93].

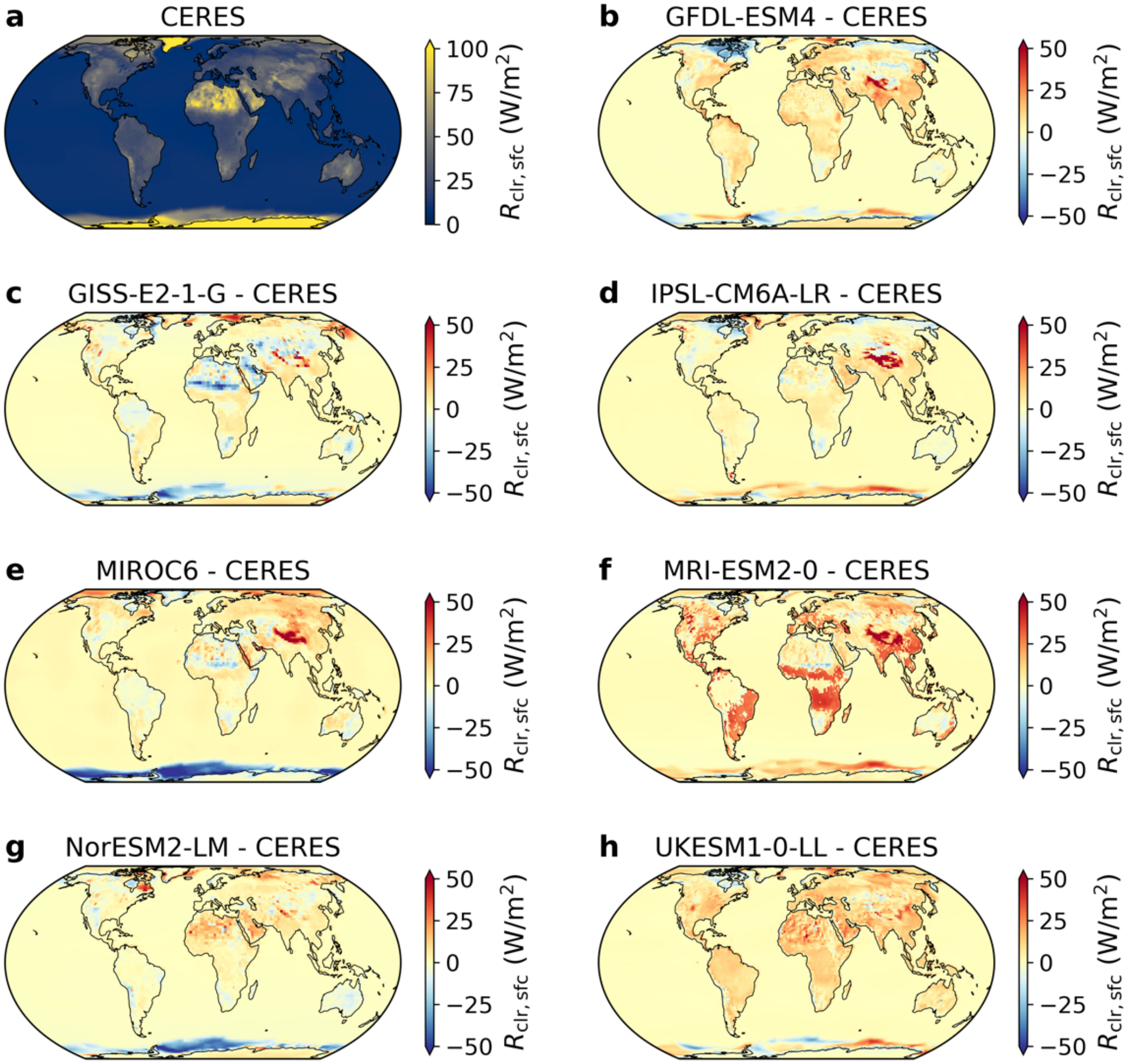

Supplementary Figure 3 | Maps of the surface contribution to clear-sky reflection for CERES and the CMIP6 models. Observed $R_{clr,sfc}$ from CERES (**a**) and the difference between the observed value and each of the CMIP6 models analyzed (**b-h**) are shown globally on an equal-area projection[88,93].

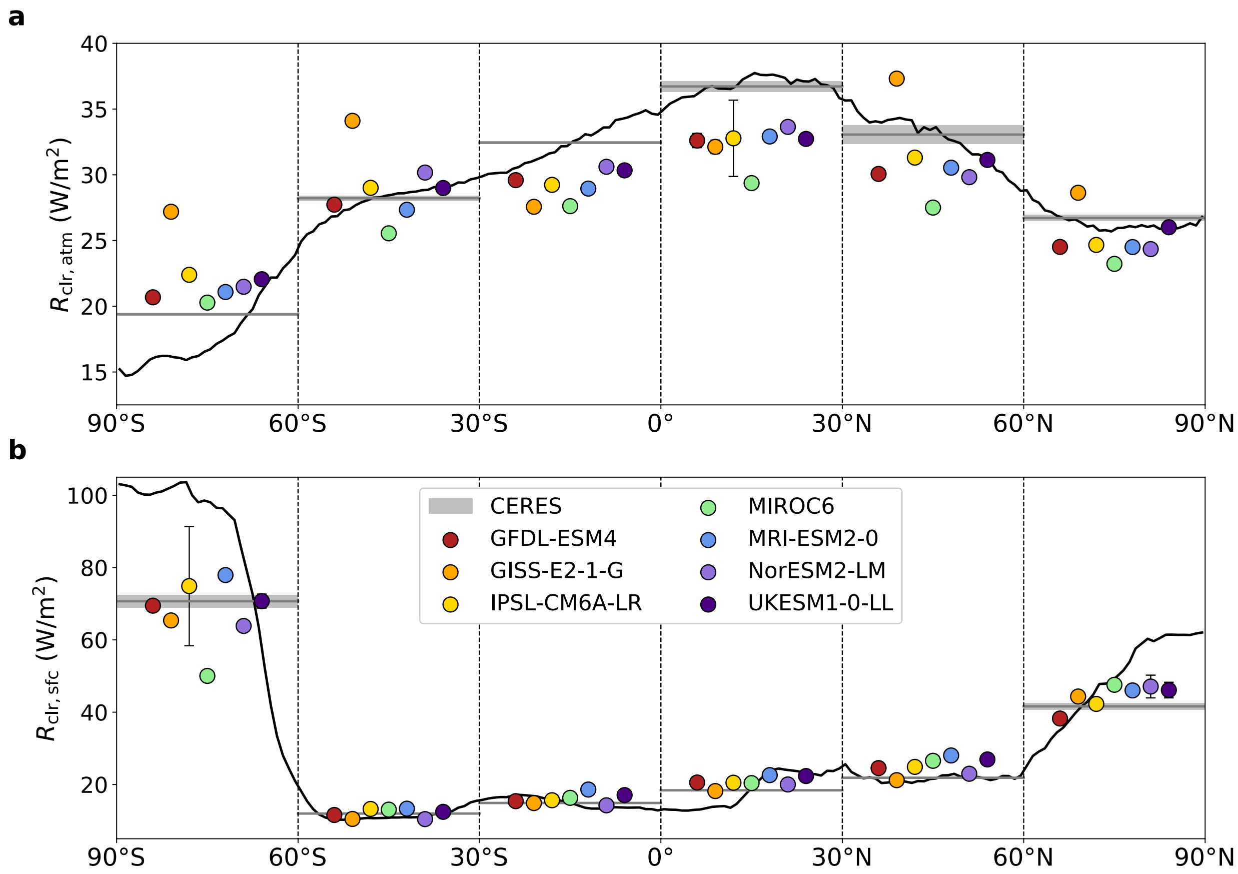

Supplementary Figure 4 | Zonal differences between CERES and the CMIP6 observations. **a** Mean CERES value and 95% confidence interval are represented by the gray line and shading and mean CMIP6 model value and 95% confidence interval are represented by the circular markers and error bars for the atmospheric component of the clear-sky reflection for the Southern Hemisphere poles (90°-60° S), midlatitudes (60°-30° S), and tropics (30° S-0°) and the Northern Hemisphere tropics (0°-30° N), midlatitudes (30°-60° N), and poles (60°-90° N). Zonal mean CERES observations are shown as a dark gray line for reference. **b** As in **a**, but for the surface component of the clear-sky reflection. Large errors for IPSL-CM6A-LR in the Northern Hemisphere tropics and Southern Hemisphere poles are primarily due to a very high degree of temporal autocorrelation as opposed to large standard deviations.

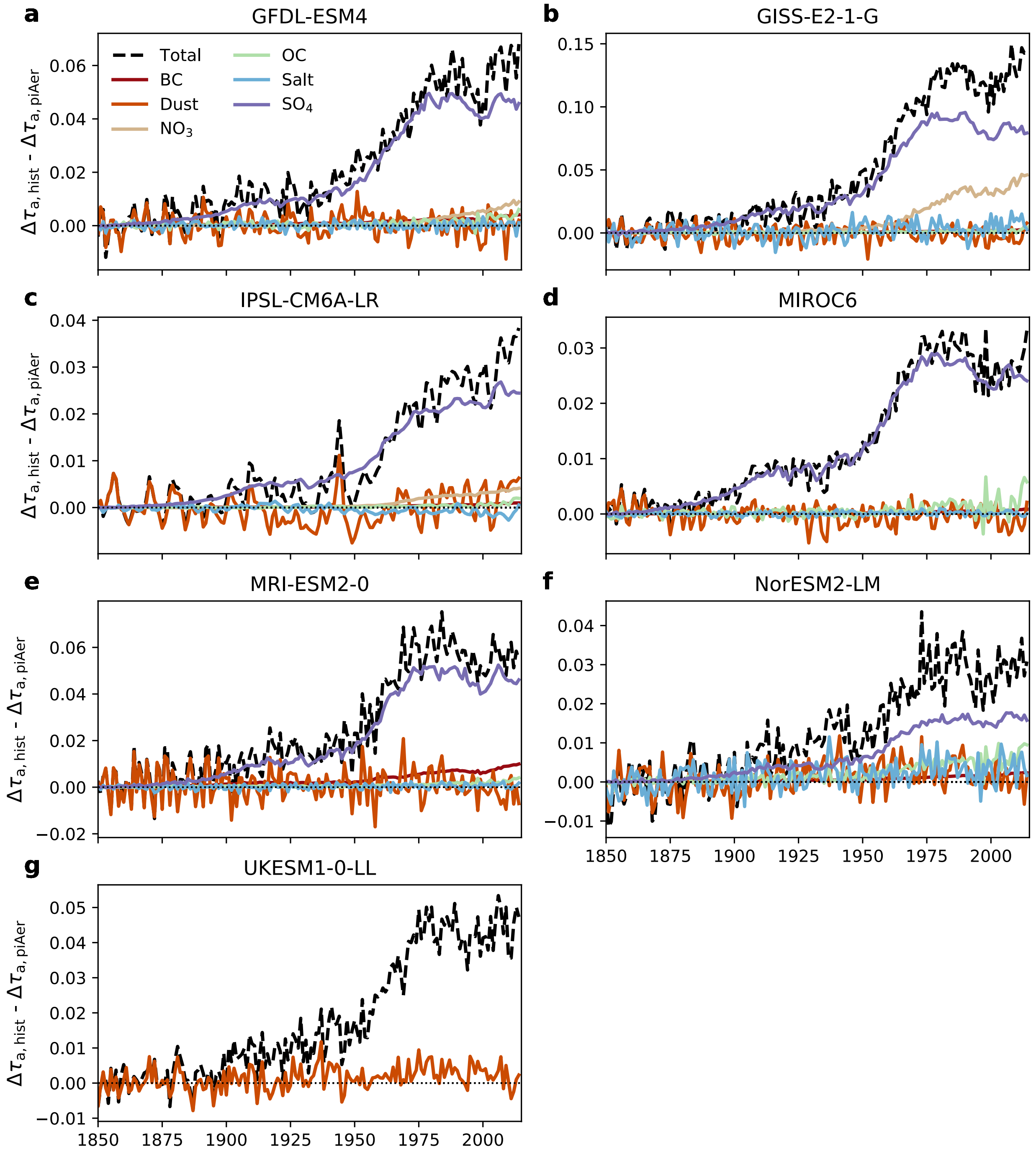

Supplementary Figure 5 | Change in hemispheric aerosol asymmetry due to historical emissions of aerosols and their precursors. Differences in the evolution of the asymmetry in total AOD and each available species [black carbon (BC), dust, nitrate (NO_3), organic carbon (OC), sea salt, and sulfate (SO_4)] between the historical ($\Delta\tau_{a,hist}$) and hist-piAer ($\Delta\tau_{a,piAer}$) simulations are shown for each model (**a-g**). Not all models contain or output statistics for all aerosol species.

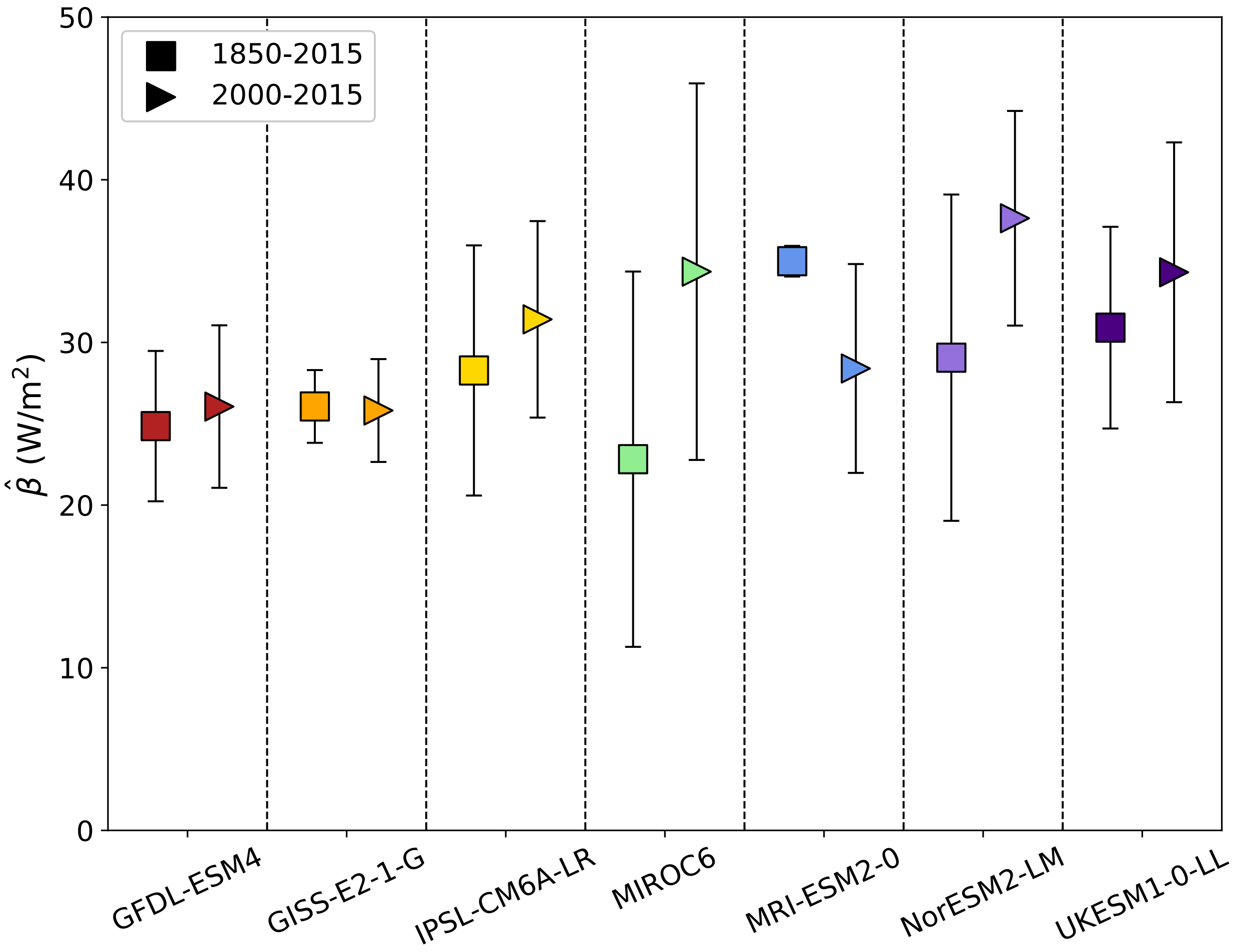

Supplementary Figure 6 | Modern and full historical regression slopes for each CMIP6 model. Regression slopes ($\hat{\beta}$, units of W/m^2 in $\Delta R_{clr,atm}$ per unit $\Delta\tau_a$) for each CMIP6 model and their 95% confidence intervals are represented by colored markers (square for the 1850-2015 regression, triangle for 2000-2015 only) and error bars.

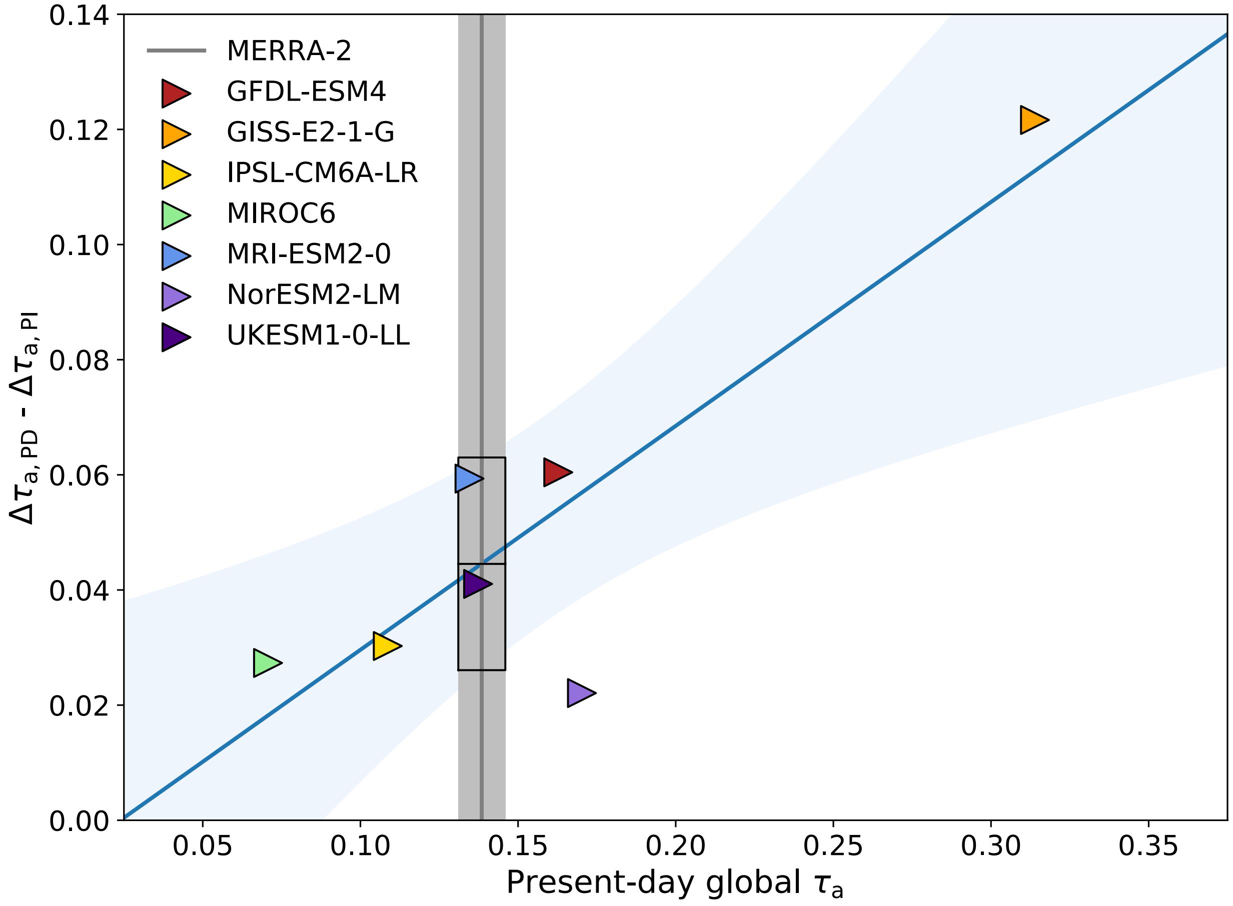

Supplementary Figure 7 | Emergent constraint for the change in hemispheric aerosol contrast from present-day to pre-industrial based on present-day global mean aerosol optical depth. CMIP6 models are represented by the colored triangles and their regression slope and its 95% confidence interval by the blue line and shading. MERRA-2 values for the present-day global mean AOD and its 95% confidence interval are represented by the gray line and shading. The constraint on the present-day to pre-industrial change in $\Delta\tau_a$ is represented by the black box, with a center line at the mean value and extent based on the 95% confidence interval.

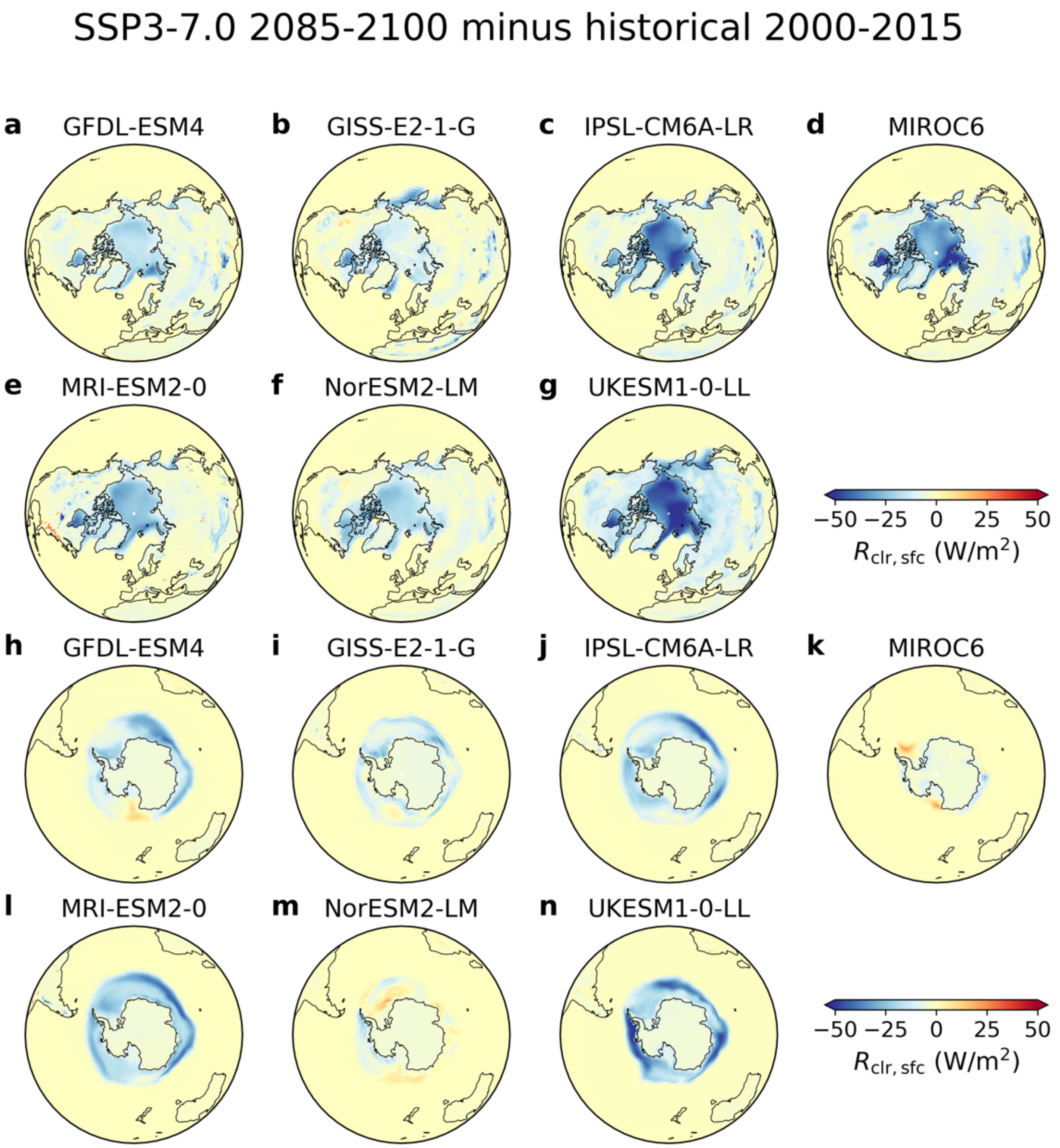

Supplementary Figure 8 | Change in surface reflection over the poles in the SSP3-7.0 high-emissions scenario. Difference in surface reflection between the SSP3-7.0 end-of-century (2085-2100 mean) and historical present-day (2000-2015 mean) for each CMIP6 model centered around the Arctic (**a-g**) and Antarctic (**h-n**) using an orthographic map projection[88].

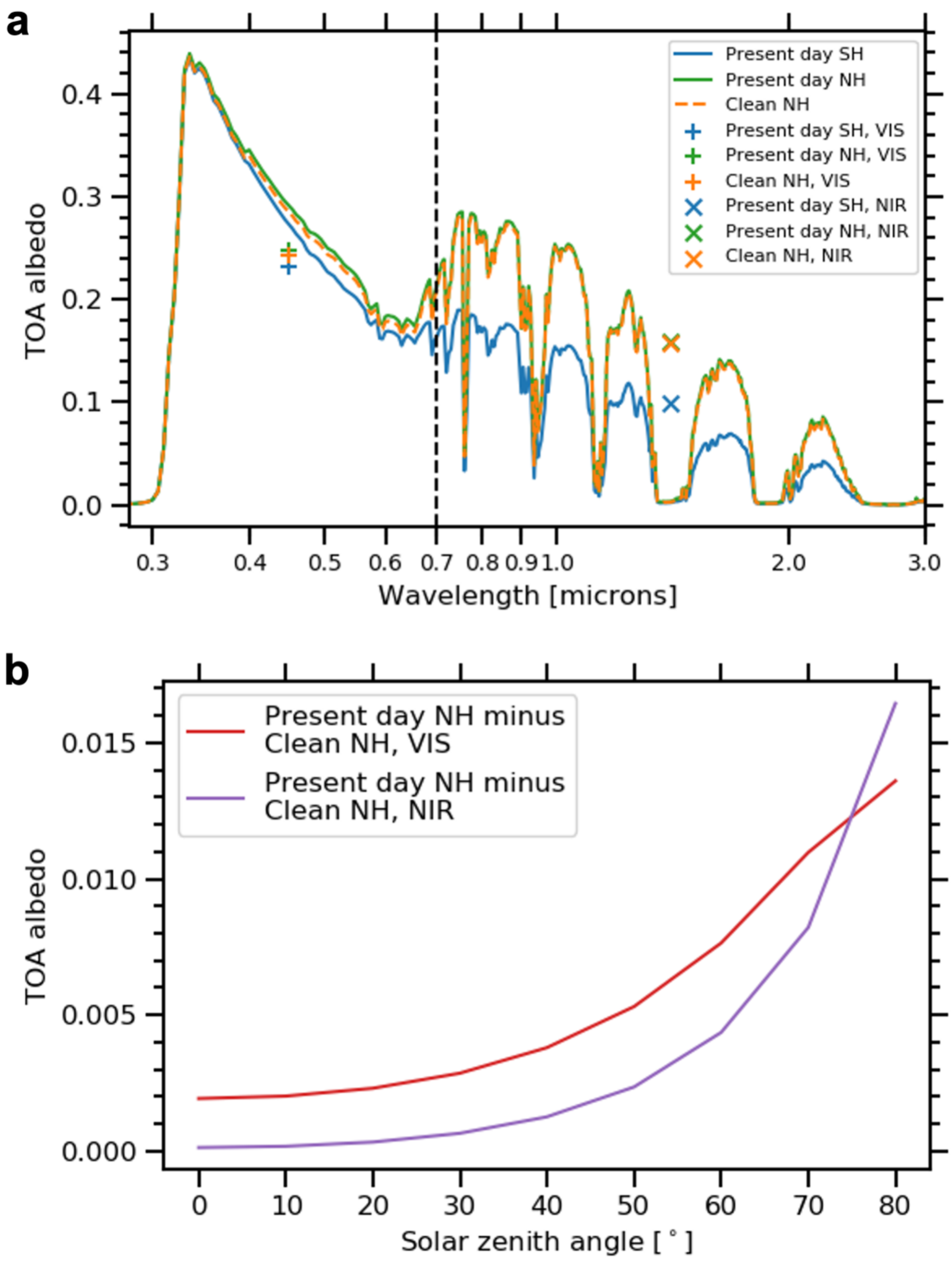

Supplementary Figure 9 | Radiative transfer calculations of the clear-sky albedo for a hypothetical "cleaner" Northern Hemisphere. a Spectral top-of-atmosphere albedo at an example solar zenith angle of 50° for present-day Norther Hemisphere, present-day Southern Hemisphere, and clean Northern Hemisphere. Spectrally-integrated albedos for the ultraviolet and visible portion (VIS) of the spectrum (0.2-0.7 μm) are represented by the colored pluses and the near-infrared portion (NIR) of the spectrum (0.7-3.0 μm) by crosses. **b** Differences between present-day and clean NH for VIS and NIR albedo as a function of solar zenith angle.
